# SIK1 suppresses colorectal cancer metastasis and chemoresistance via the TGF-β signaling pathway

**DOI:** 10.7150/jca.83708

**Published:** 2023-08-06

**Authors:** Yuan Gao, Hongming Li, Ping Wang, Junjiang Wang, Xueqing Yao

**Affiliations:** 1 The Second School of Clinical Medicine, Southern Medical University, Guangzhou 510000, China.; 2 Department of Gastrointestinal Surgery, Department of General Surgery, Guangdong Provincial People's Hospital, Guangdong Academy of Medical Sciences, Southern Medical University, Guangzhou 510000, China.; 3 Department of General Surgery, Guangdong Provincial People's Hospital Ganzhou Hospital (Ganzhou Municipal Hospital), Ganzhou, 341000, China.; 4 Department of Colorectal surgery, Guangdong Provincial Hospital of Chinese Medicine, Guangzhou, 510120, China.

**Keywords:** salt-induced kinase 1 (SIK1), colorectal cancer, TGF-β/Smad signaling pathway, epithelial-mesenchymal transition, oxaliplatin resistance

## Abstract

In the present study, we investigated the role of salt-induced kinase 1 (SIK1), a serine/threonine kinase protein, in colorectal cancer (CRC). Despite the reported association of SIK1 with tumor malignancy suppression in various cancers, limited research has been conducted on its function in CRC. Our findings revealed that SIK1 expression was low in CRC cells. The results of a KEGG pathway analysis showed a strong association between SIK1 and the TGF-β signaling pathway. In addition, a coimmunoprecipitation assay validated the interaction between SIK1 and Smad7. Our data indicate that SIK1 inhibited the phosphorylation of Smad2, a critical molecule in the Smad-related TGF-β pathway, and downstream target genes of the TGF-β pathway. Furthermore, SIK1 was found to inhibit indicators of epithelial-mesenchymal transition (EMT) and reverse oxaliplatin resistance in CRC. Additionally, SIK1 reduced cell migration and invasion. Our results suggest that the inhibitory effect of SIK1 on the TGF-β pathway contributes to the suppression of metastasis and oxaliplatin chemoresistance in CRC. However, this effect was reversed by galunisertib (LY2157299). In conclusion, our findings provide novel insights into the role of SIK1 in the regulation of the TGF-β pathway in CRC, suggesting its potential as a therapeutic target for the treatment of CRC. Further studies are required to fully characterize the mechanism underlying these observations and to validate these findings in animal models.

## 1. Introduction

Colorectal cancer (CRC) is the third most common cancer, with an increasing incidence in recent years 1. Despite improvements in treatment for CRC, 5-year overall survival and disease-free survival are still low in patients with locally advanced or metastatic colorectal cancer 2. Surgery, chemotherapy, and radiation can control various local tumors, but the overall utility of these treatments in limiting the ability of tumors to metastasize is not optimistic 3. Therefore, there is an urgent need to identify prognostic markers and therapeutic targets for metastatic and drug-resistant tumors.

SIK1 is a serine/threonine kinase protein. It is involved in cell cycle regulation, gluconeogenesis, lipid regulation, muscle growth, and differentiation 4, 5. It has been reported to be associated with tumor malignancy suppression in a variety of cancers, of which SIK1 has been comprehensively reported. SIK1 is involved in the regulation of tumor suppression in lung cancer 6, 7. In addition, SIK1 knockdown can increase metastasis by increasing Epithelial-Mesenchymal Transition (EMT) in CRC cells 8, 9, suggesting that SIK1 may be a tumor suppressor gene.

Transforming growth factor-β (TGF-β) induces numerous pleiotropic pathways, which are regulated by the cellular environment and integrated with different signaling pathways 10. In cancer, the pleiotropic response to TGF-β leads to a variety of gene responses, ranging from apoptotic suppression of tumor responses in early-stage tumors to proliferation, invasion, angiogenesis, and carcinogenesis responses in advanced cancers 11, which are closely related to the regulation of EMT, and EMT can promote the transformation of tumor cells into cancer stem cells (CSCs) and acquire resistance characteristics to multiple drugs. The main research direction of SIK1 regulating the TGF-β signaling pathway is further in non-N-cadherin diseases 12, 13, but there is a lack of research in cancer, and even the specific mechanism has not been determined and still needs to be researched in depth.

Therefore, the current research aimed to test the hypothesis that SIK1 plays an antineoplastic role by inhibiting the activation of the TGF-β signaling pathway and then inhibiting EMT and oxaliplatin resistance, accordingly providing a different therapeutic target for CRC.

## 2. Results

### 2.1 The expression of SIK1 is downregulated in CRC tissues and CRC cell lines

**Figure 1:**
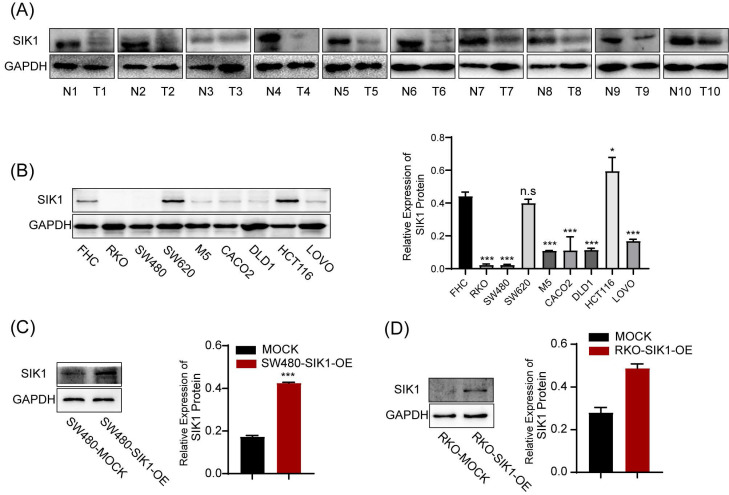
** The expression of SIK1 is downregulated in CRC cell lines.** (A) SIK1 protein expression in RKO, SW480, M5, SW620, CACO2, DLD1, HCT116, and LOVO CRC cells compared with that in immortalized colonic FHC cells by Western blotting. (B, C) Western blot analysis was performed for SIK1 in SW480 and RKO cells transfected with SIK1 overexpression virus. Statistical analysis was performed in triplicate experiments via unpaired Student's t test. Data are expressed as the mean ± SD (n=3), *n.s. p*>=0.05, *p<0.05, **p<0.01, *** p<0.001.

SIK1 expression was generally reduced in CRC tissues (Figure 1A), and generally downregulated in CRC cell lines. Among them, RKO and SW480 were significantly downregulated (both, p<0.001), and HCT116 was the most upregulated cell line (Figure 1B). Subsequently, RKO and SW480 cell lines overexpressing SIK1 were constructed and generated stable transfectants, and the transfection efficiency was verified by western blotting (both, p<0.001) (Figure 1C, D). Furthermore, we produced a data analysis from the TCGA database about the promoter methylation level of SIK1 in CRC, and we found that compared with normal tissues in the colon, CRC tissue exhibited more promoter methylation of SIK1 (*p*<0.001) (Figure S1A). In addition, TCGA database analysis showed that SIK1 promoter methylation level was significantly increased in colorectal cancer tissues (Figure S1A). However, the analysis of the Gene Expression Profiling Interactive Analysis (GEPIA) database indicated no prognostic value associated with SIK1 expression in The Cancer Genome Atlas (TCGA) CRC cohorts (Figure S1B). In our cohort, the expression of SIK1 did not significantly differ between tumor and adjacent non-tumor tissue (Figure S1C).

### 2.2 SIK1 inhibits the metastatic ability of CRC cells

**Figure 2:**
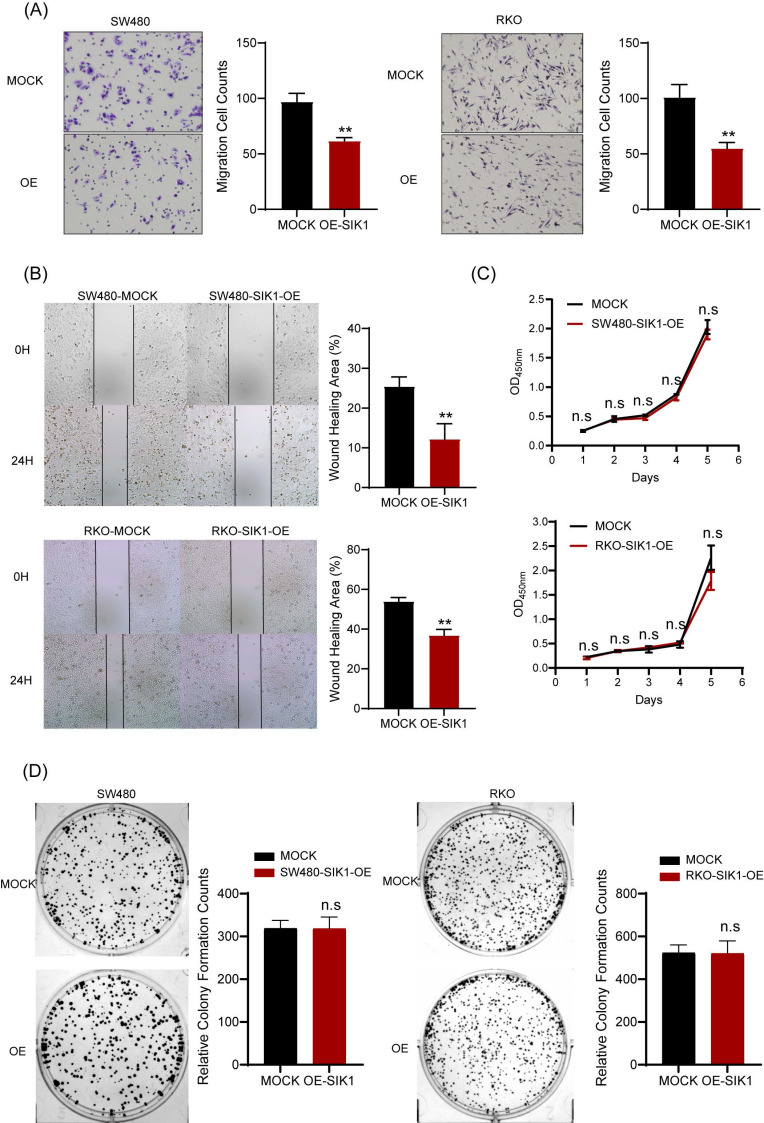
** SIK1 can inhibit the metastatic ability of CRC cells.** (A) Transwell assays were employed to examine cell migration. (B) Wound-healing assay was used to detect the cell migration and invasion capabilities of SW480 and RKO cells after transfection with SIK1. (C, D) Effect of SIK1 on cell proliferation by CCK-8 assay and colony formation assay. Statistical analysis was performed in triplicate experiments via unpaired Student's t test. Data are expressed as the mean ± SD (n=3), *n.s. p*>=0.05, *p<0.05, **p<0.01, *** p<0.001.

To determine the phenotypic changes of SIK1 in CRC cell lines in vitro, a transwell migration assay and wound-healing assay were performed. The results showed that upregulation of SIK1 significantly reduced the migration and ability of the CRC cell lines RKO and SW480 (both, p<0.001, Figure 2A) and wound-healing ability (both, p<0.01, Figure 2B). In terms of detecting cell proliferation, CCK-8 and clone formation assays were performed, and the results showed that there were no significant differences in either cell proliferation or clone formation in CRC cell lines (both p>0.05, Figure 2C, D).

### 2.3 Knockdown of SIK1 promotes the migration and oxaliplatin resistance of HCT116 CRC HCT116 cells

**Figure 3:**
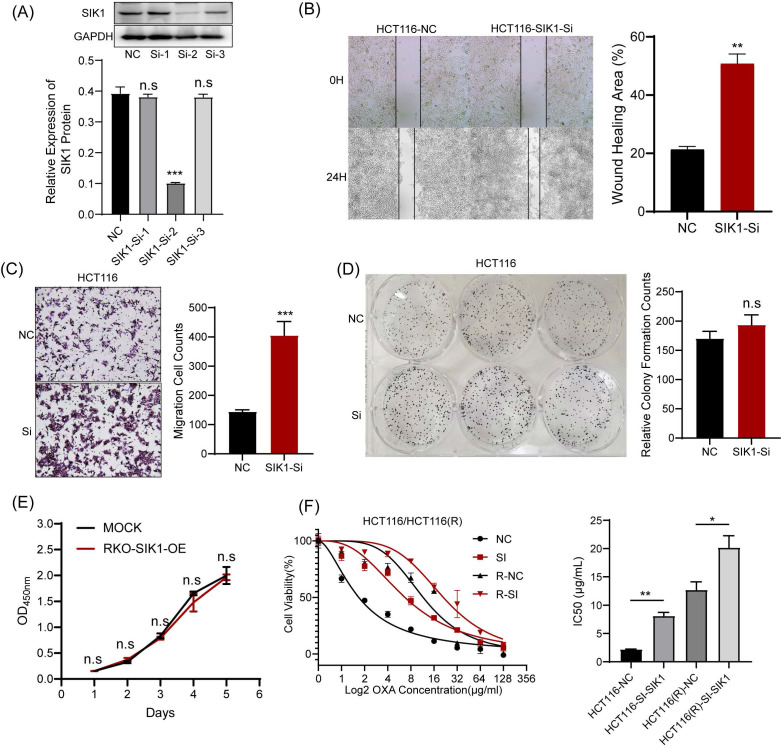
** Knockdown of SIK1 can promote the migration ability of HCT116 CRC cells.** (A) Western blot analysis was performed for SIK1 in HCT116 cells transfected with SIK1-Si-1, SIK1-Si-2, and SIK1-Si-3. Effect of SIK1 knockdown on cell migration by (B) transwell assay and (C) wound-healing assay. Effect of SIK1 knockdown on cell proliferation by (D) colony formation assay and (E) CCK-8 assay. (F) The knockdown of SIK1 significantly decreased sensitivity to OXA in normal CRC cell lines (HCT116) and OXA-resistant cell lines (HCT116(R). Statistical analysis was performed in triplicate experiments via unpaired Student's t test. Data are expressed as the mean ± SD (n=3), *n.s. p*>=0.05, *p<0.05, **p<0.01, *** p<0.001.

For functional verification against interference transfection, the experimental results can be used as a supplement to SIK1 overexpression. HCT116 was chosen for further analysis as the low-expression SIK1 cell line, and a western blot assay was performed to determine the transfection efficiency. Among them, SI-2 had the best interference performance (p<0.05) (Figure 3A). Migration and wound-healing ability were significantly increased after SIK1 knockdown (both, p<0.01, Figure 3B, C). Like the overexpression group, CCK-8 and clone formation assays were performed, and there was no significant difference (both, p>0.05) (Figure 3D, E). To further determine whether SIK1 was correlated with chemotherapy resistance in CRC, we constructed an OXA-resistant HCT116 cell line (HCT116R), and a cytotoxicity assay was conducted. The IC50 of OXA in siSIK1-transfected cancer cells was significantly higher than that in siNC-transfected cells, both in regular cancer cell lines and OXA-resistant cell lines (both, p<0.01, Figure 3F). In addition, another SIK1-high-expressing cell line SW620 and OXA-resistant SW620 cell line (SW620R) were selected for OXA drug IC50 assay, and the results were like HCT116 and HCT116(R) (Figure S2A, B).

### 2.4 SIK1 may regulate the phenotypic changes of CRC cells through the TGF-β signaling pathway

**Figure 4:**
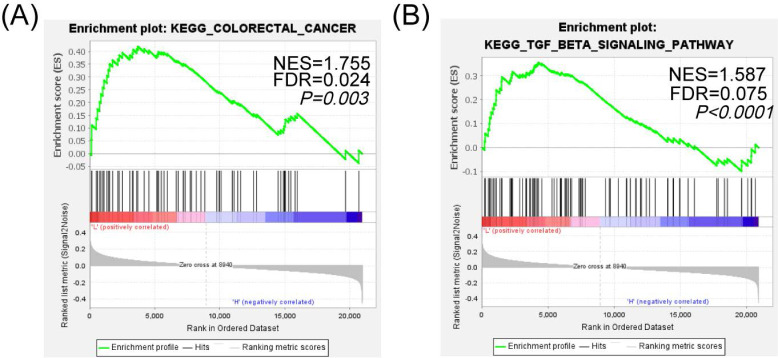
** SIK1 may regulate the phenotypic changes of CRC cells through the TGF-β signaling pathway.** (A) GSEA KEGG pathway enrichment analysis in colorectal cancer. (B) GSEA KEGG pathway enrichment analysis in the TGF-β signaling pathway.

To further analyze the specific pathway of SIK1 regulation in colon cancer, KEGG pathway analysis of GSEA (Version GSEA_4.2.0) was performed, and the data were obtained from the GEO database (GEO, GSE101896). The results showed that the 'KEGG_COLORECTAL_CANCER' pathway was enriched in the SIK1 downregulated group (p=0.003, FDR=0.024, NES=1.755, Figure 4A), and it was significantly enriched in the TGF-β signaling pathway (p<0.0001, FDR=0.075, NES=1.587, Figure 4B). In addition, the low expression of SIK1 may be related to the enrichment of the P53 pathway (p=0.009, FDR=0.086, NES=1.556, Figure S3). In addition, GEPIA bioinformatics analysis showed that SIK1 was positively correlated with SMAD7 (p=0.0036, R=0.2, Figure S4).

### 2.5 SIK1 regulates the downstream target genes of the TGF-β pathway and EMT

**Figure 5:**
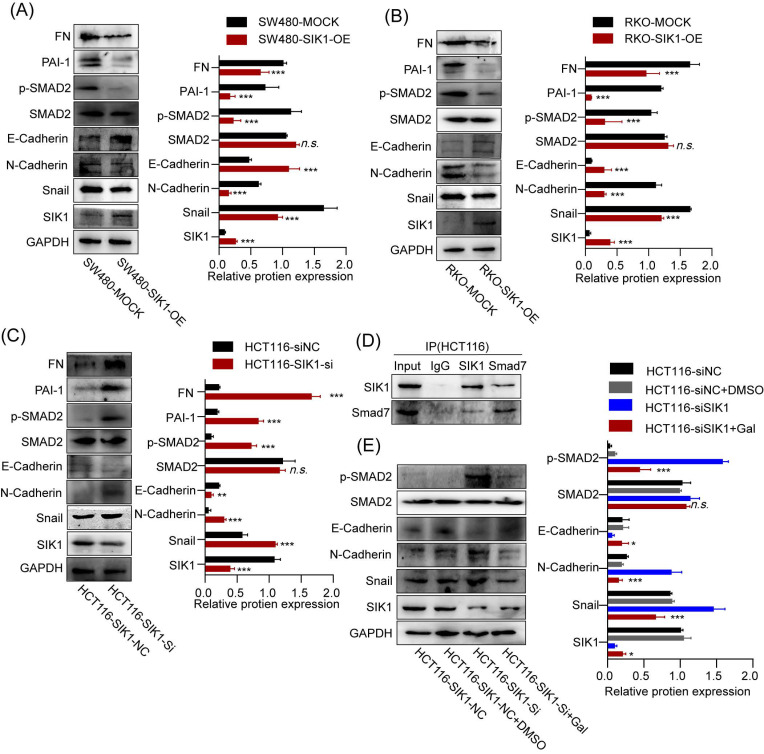
** SIK1 can regulate the downstream target genes of the TGF-β pathway and EMT.** (A, B) The protein expression of SW480 and RKO cells after transfection with SIK1 by western blot. (C) The protein expression of SIK1 knockdown on the TGF-β pathway and EMT by western blot. (D) Co-IP experiments were performed using either an anti-SIK1 antibody to pull down Smad7 or an anti-Smad7 antibody to pull down SIK1 in HCT116 cells to identify an interaction between SIK1 and Smad7. (E) Western blot analyses were performed for E-cadherin, N-cadherin, Snail, PAI-1, FN, SMAD2, pSMAD2, and SIK1 in the indicated cells between SIK1 knockdown and SIK1 knockdown with the TGF-β pathway inhibitor LY2157299 (Gal). Statistical analysis was performed in triplicate experiments via unpaired Student's t test. Data are expressed as the mean ± SD (n=3), *n.s. p*>=0.05, *p<0.05, **p<0.01, *** p<0.001.

To investigate whether SIK1 regulates the malignant phenotype of colorectal cancer through the TGF-β signaling pathway, a western blot assay was performed. As shown in Figure 5A and B, the expression of pSMAD2, FN, and PAI-1, TGF-β pathway genes, was significantly downregulated in the RKO and SW480 cell lines after SIK1 overexpression (p<0.01), and the expression of pSmad2, FN, and PAI-1 was upregulated in the HCT116 cell line after SIK1 knockdown (p<0.01, Figure 5C). In addition, to study the change in EMT of SIK1 knockdown of the TGF-β signaling pathway, western blot analyses were performed for E-cadherin, N-cadherin, and Snail in the indicated cells transfected with SIK1 or SIK1 knockdown. As shown in Figure 5A, B, N-cadherin and Snail were downregulated (both, p<0.01), and E-cadherin was upregulated after transfection with SIK1. However, N-cadherin and Snail were upregulated, and E-cadherin was downregulated after SIK1 knockdown (both, p<0.01, Figure 5C).

### 2.6 SIK1 regulates the TGF-β signaling pathway by binding to the signal inhibitory molecule Smad7

Expression of SIK1 was positively correlated with SMAD7, p<0.001, (Fig. S3) via the analysis of GEPIA database. To further investigate the binding between SIK1 and Smad7, co-IP experiments were performed. The co-IP results showed an interaction between Smad7 and SIK1 in RKO and SW480/SIK1 cells (p<0.0001, Figure 5D). In addition, we selected galunisertib (LY2157299), an inhibitor of TGFBR1 in the TGF-β pathway, and FN, PAI-1, E-cadherin, N-cadherin, and Snail were detected by western blotting. From Figure 5H, the results showed that compared with the HCT116NC group, the expression of FN and PAI-1 in HCT116 cells with SIK1 knockdown increased (both, p<0.01), and the expression of N-cadherin and Snail increased (both, p<0.01), while E-cadherin expression decreased (p<0.05). Compared with the HCT116 cells in the SIK1 knockdown group, the HCT116 cells in the SIK1 knockdown with LY2157299 treatment group showed decreased expression of FN and PAI-1 (both, p<0.01), decreased expression of N-cadherin and Snail (both, p<0.01), and increased E-cadherin expression (p<0.01).

### 2.7 The regulation of SIK1 in HCT116 CRC cells could be rescued by LY2157299

**Figure 6:**
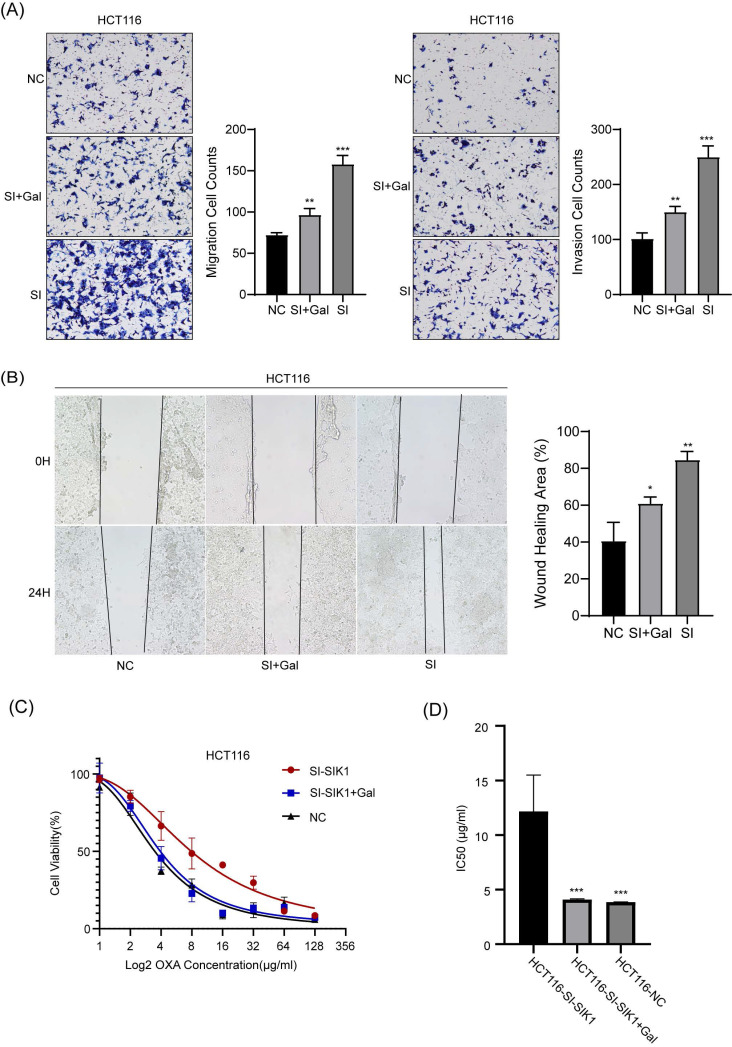
** The regulation of SIK1 in HCT116 CRC cells could be restored.** (A) Effect of SIK1 knockdown with LY2157299 treatment on cell migration and invasion by Transwell assay. (B) Effect of SIK1 knockdown with LY2157299 treatment on cell migration and invasion capabilities by wound-healing assay. Data are expressed as the mean ± SD (n=3), *n.s. p*>=0.05, *p<0.05, **p<0.01, *** p<0.001.

We conducted recovery experiments through transwell and wound-healing assays. As shown in Figure 6 A, compared with the HCT116-SIK1-NC group, the HCT116-SIK1-SI group exhibited greater invasion and migration abilities (both, p<0.001). Compared with the HCT116-SIK1-SI group, HCT116-SIK1-SI with LY2157299(Gal) treatment restored invasion and migration (both, p<0.01). The wound healing of the HCT116-SIK1-SI group was significantly enhanced compared with that of the HCT116-SIK1-NC group (p <0.001, Figure 6B). Compared with the HCT116-SIK1-SI group, the wound healing ability of HCT116-SIK1-SI with LY2157299 treatment was restored (p<0.001, Figure 6B). This phenomenon was shown in the cytotoxicity assay of OXA treatment, and the OXA resistance of SIK1 downregulation could be restored by LY2157299 treatment (p<0.001, Figure 6C).

### 2.8 Downregulation of SIK1 reverses chemotherapy resistance in vivo

**Figure 7:**
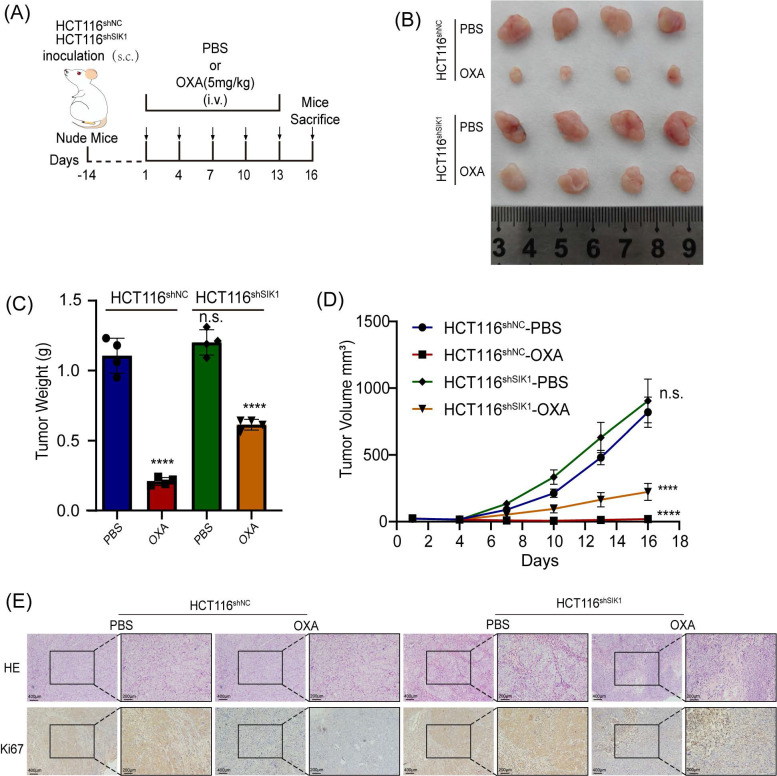
** Downregulation of SIK1 reverses chemotherapy resistance *in vivo*.** (A) Schematic illustration of the treatment schedule and tumor evaluation. Stable transfection of shSIK1 cell lines was established and injected into nude mice subcutaneously. (B) Images of the resected tumors of HCT116shNC or HCT116shSIK1 tumor-bearing nude mice. (C) The weight of the tumors. ****P < 0.0001. (D) The tumor inhibition profiles of the mice receiving different treatments. Mean ± SD (n = 4). ****P < 0.0001. (E) Representative HE and Ki67 IHC staining images among the groups. Scale bar = 400 μm for 100× magnification images and 200 μm for 200× magnification images. Data are expressed as the mean ± SD (n=3), *n.s. p*>=0.05, *p<0.05, **p<0.01, *** p<0.001.

To further investigate the effect of SIK1 on the chemotherapy resistance of CRC *in vivo*, stable transfection of SIK1-downregulated HCT116 cells and NC cells was established by lentivirus. HCT116shSIK1 and HCT116shNC cells were injected subcutaneously into the left flanks of mice. The mice received intravenous injection (*i.v.*) of OXA and PBS six times after the tumor was less than 150 mm3 HCT116shSIK1. The protocol of the in vivo experiments is shown in Figure 7A, and the images of the resected tumors of the HCT116shNC or HCT 116shSIK1 tumor-bearing nude mice are shown in Figure 7B. SIK1 knockdown significantly increased in vivo chemotherapy resistance, leading to decreased efficacy, tumor weight, and tumor volume in HCT116 nude mice (Figure 7B, C and D). All the mice survived at the end of the treatment, and the tumor was surgically resected and stained with HE and IHC of Ki67 for further analysis. When SIK1 was downregulated combined with OXA chemotherapy, the proliferative ability of SIK1 downregulated combined with OXA chemotherapy was stronger than that of the shNC+OXA chemotherapy group, and the expression of Ki67 was higher (Figure 7E). The above experiments indicated that SIK1 may be an ideal target to enhance the therapeutic efficiency of chemotherapy *in vivo*.

## 3. Discussion

In recent years, it has been found that SIK1 can inhibit the development of tumors, has obvious abnormal expression in a variety of tumors, and has an obvious correlation with the pathological stage and prognosis of tumors 14. It has been reported in the literature that reduced SIK1 expression is associated with poor prognosis in ovarian cancer patients. SIK1 knockdown can induce interstitial cell transformation and metastasis to the lung, suggesting that SIK1 may be a tumor suppressor gene 15. It has been reported that pancreatic cancer cells with SIK1 knockdown expression are more likely to invade and metastasize and are related to the regulation of microRNA-203 (miR-203). The expression of SIKl in pancreatic and paracancerous tissues was significantly lower than that in paracancerous tissues 16. The expression of SIKl in gemcitabine-resistant pancreatic cancer cell lines was significantly lower than that in sensitive cell lines. SIKl inhibited the expression of c-myc, CDKl, and Ki-67 and promoted the expression of p53 and p21 5, 17, 18. In addition, SIK1 knockdown has been reported to induce EMT, and these results suggest that SIK1 may be a tumor suppressor 19, 20. However, none of these studies investigated the role and mechanism of SIK1 in CRC metastasis and chemoresistance. In our early study, distant metastasis was still found after radical surgery for colorectal cancer (CRC), and gene sequencing analysis of tumor samples from CRC patients revealed that liver metastases were associated with genetic mutations in primary tumors 21.

Additional in vitro studies have shown that SIK1 has a significant inhibitory effect on the metastatic function of CRC and inhibits EMT in CRC. EMT is gradually being considered a novel tumor resistance mechanism. Tumor cells develop specific drug resistance tendencies during EMT progression 22. Several reports have shown that EMT is related to chemotherapy resistance 23, 24. The specific mechanism is that EMT can promote the transformation of cancer cells into cancer stem cells (CSCs) and acquire drug resistance 22, 25, and we found that SIK1 can inhibit oxaliplatin resistance in oxaliplatin drug experiments and in vivo experiments.

To further analyze the mechanism of action of SIK1 in CRC, we selected the data in the GEO database (GSE101896) for KEGG pathway analysis in GSEA software, and the results showed that the main enrichment pathway of SIK1 in CRC was the TGF-β signaling pathway. In addition, in vitro experiments were conducted to verify that this pathway was activated after SIK1 knockdown. However, as our in vitro functional experiments found that it had no significant effect on tumor proliferation function, the reason was investigated. In addition to activation of the TGF-β pathway, the downregulation of SIK1 is also associated with the activation of the P53 pathway. It is well known that the P53 pathway, as a tumor suppressor pathway, inhibits tumor proliferation and enhances tumor apoptosis 26. Until now, due to the complexity of tumors and the limitations of single gene analysis, the same gene has different functional manifestations, or even functional counteractions, in different tumor cells and even in different physiological and pathological environments.

In cancer, pleiotropic responses to TGF-β lead to a diverse array of genetic responses, ranging from cytotoxic and apoptotic tumor suppressor responses in early tumors to proliferation, invasion, angiogenesis, and carcinogenesis in advanced cancers 11. In addition, the downstream target genes of TGF-β, FN, and PAI-1 also promote tumor metastasis 27, 28. Furthermore, there are two main ways in which TGF-β performs its various functions. One involves Smads: TGF-β binds to the TGF-β receptor (TGFBR1/2) and further phosphorylates Smad2/3, forming a complex with Smad4 in the nucleus for gene regulation 29. However, Smad family members are also involved in blocking the TGF-β pathway. For example, Smad7 can bind to TGFBR1 and block the binding of TGF-β to further phosphorylate Smad2/3 and form a complex with Smad4 to carry out a series of gene regulations 30. Another form does not involve Smads: It involves direct phosphorylation of PAR6, the cellular polarity regulator, by TGFBR2 and the activation of TGF-β receptors and pathways such as TAK1 and JNK 31-33. TGF-β is a well-coordinated process with EMT, in which epithelial cells lose cell connectivity and polarity and transform into mesenchymal cells capable of migration and invasion 34. Molecularly, this process is accompanied by a cell connection switch from E-cadherin to N-cadherin 35, 36. TGF-β signaling is known to induce important molecules of EMT in a Smad4-dependent manner by inducing translocation of the SMAD4/2/3 complex to the nucleus, leading to the expression of the mesenchymal markers snail, slug, Twister, and ZEB 37, 38.

It has been reported that in nontumor diseases, such as glomerular fibrosis, SIK1 inhibits the phosphorylation of Smad2 mainly by binding to Smad7, a TGF-β pathway inhibitor, and subsequently fails to bind to Smad3, completely translocates to the nucleus, and activates downstream pathways 39, 40. Subsequently, we performed Co-IP experiments to verify the binding between SIK1 and Smad7, as well as the regulation of the downstream genes FN, PAI-1, and EMT. In addition, the effect of SIK1 knockdown on the TGF-β pathway in CRC can be rescued by the TGF-β pathway inhibitor LY2157299. As discussed above, SIK1 can suppress colorectal cancer metastasis and oxaliplatin resistance via TGF-β signaling pathway-mediated EMT.

Although a functional experiment of SIK1 was conducted in this study, and it was found that SIK1 was related to the metastasis of CRC and that it had little effect on proliferation, the internal mechanism of this phenomenon was not investigated deeply. In addition, although this paper revealed the relationship between SIK1 and the TGF-β signaling pathway in CRC and found its relationship with EMT and OXA drug resistance, the study of whether there is an interaction between EMT and OXA drug resistance has not been in depth, although there is literature supporting the potential correlation between EMT and OXA. However, more research is needed to confirm a direct relationship. Next, according to the phenomenon found in this study, the specific mechanism of interaction between EMT and OXA resistance in CRC will be studied.

## 4. Materials and methods

### 4.1 Patient samples and cell culture

In total, 10 pairs of CRC tissue and adjacent normal tissue were collected from patients after surgery at the Guangdong Provincial People's Hospital. All patients signed informed consent prior to tissue collection and were diagnosed with CRC by pathological examination. The inclusion criteria of CRC patients were undergoing surgical resection and were pathologically diagnosed with adenocarcinoma. The study was approved by the ethics committee of Guangdong Provincial People's Hospital Affiliated under the grant number of GDREC2019504H and 2020-205H-3. The normal human colorectal immortalized cell line FHC and CRC cell lines RKO, SW480, M5, SW620, CACO2, DLD1, HCT116, and LOVO were obtained from NCACC (National Collection of Authenticated Cell Cultures). Cell culture was performed in RPMI 1640 medium (Sigma Aldrich, United States) supplemented with 10% fetal bovine serum (FBS, Gibco BRL, United States) at 37 °C and 5% CO_2_. The OXA-resistant cell lines HCT116R and SW620R were purchased from Meixuan Bioscience Inc. (Shanghai, China). The OXA resistance cell line HCT116 was purchased from Meixuan Bioscience Inc. (Shanghai, China). OXA-resistance SW620 cell lines were generated by exposure to gradually increasing concentrations of OXA. SW620 cells were separately treated with OXA at an initial concentration less than the IC50. When cells adapted to that concentration, the OXA concentration was increased to 15 μg/mL. Through this process, OXA-resistant cell lines were established. The OXA resistance cell lines were designated as HCT116(R) and SW620(R). The morphology did not change significantly after the cell lines acquired the chemoresistance.

### 4.2 Quantitative real time-PCR (qRT-PCR)

TRIzol (Thermo Fisher Scientific, USA) was utilized for total RNA extraction, followed by reverse transcription into cDNA via reverse transcriptase. Real-time PCR was carried out with the DNA fluorescent dye SYBR Green I at 60 °C for 1 min, 95 °C for 15 and 72 °C for 30 s for 40 cycles, and 95 °C for 5 min. The following Th2 primer sequences were used for qPCR (5'-3'): GAPDH Forward Primer TCAACGGATTTGGTCGTATTGGGCG, Reverse Primer CTCGCTCCTGGAAGATGGTGATGGG; SIK1 Forward Primer GAGTCACCAAAACGCAGGTTG, Reverse Primer AGTGACGATGTAAAGCATGTCC; PAI-1 Forward Primer ACCTCTGAGAACTTCAGGATGC, Reverse Primer GGGTGAGAAAACCACGTTGC; FN Forward Primer CCGCCGAATGTAGGACAAGA, Reverse Primer GTGTAGGGGTCAAAGCACGA; All reactions were performed in triplicate, and the data were analyzed using comparative 2^-ΔΔCT^ methods.

### 4.3 Cell transfection

Three siRNAs (GeneChem, China) were designed to silence SIK1 *in vitro,* and finally, the most effective siRNA sequence was selected for further analysis. HCT116 cells were inoculated in 12-well plates with 2 mL of cell suspension in each well and cultured in an incubator for 24 h at 37 °C and 5% CO_2_. Then, HCT116 cells were transfected with small interfering ribonucleic acid (siRNA) targeting human SIK1 or NC (negative lentivirus group). The transfection method was performed according to the Lipofectamine 3000 (Invitrogen, Carlsbad, CA, United States) transfection reagent instructions. The cells incubated for 24 h were collected for subsequent experiments. RKO and SW480 cells were transfected with lentivirus targeting human SIK1 (GeneChem, China), and the experimental groups were as follows: MOCK (negative control group) and SIK1-OE (overexpression group). Western blotting was used to detect the transfection efficiency 24 h and 48 h after transfection, and real-time polymerase chain reaction was used for verification.

### 4.4 Cell viability assay

To investigate the cell viability of the cell lines, the cell counting kit 8 (CCK-8) detection kit (Thermo Fisher Scientific, China) was utilized in our study. The cell suspension (100 μl per well, approximately 1000 cells/100 μl) was injected into 96-well reaction plates, and then 10 μl of CCK-8 solution was added to each well for an additional incubation for 2 h. The optical density was measured at 450 nm for each sample using an enzyme-labeled instrument.

### 4.5 Colony formation assay

A total of 1,000 cells were plated on 60 mm dishes in full medium (10% FBS in RPMI 1640). After 14 days, cells derived from the dishes were washed with PBS, fixed for 10 mins in 4% paraformaldehyde (Biosharp, China) at room temperature, and stained for 20 mins with 0.25% crystal violet (Sigma, United States). Plates were washed and imaged, and analysis of colony number and colony size was performed using ImageJ software.

### 4.6 Transwell assay

The cells at a density of 10^5^ cells/ml were digested with trypsin-EDTA to prepare a cell suspension (200 μL), which was seeded into the upper chamber of Matrigel-coated or uncoated Transwell chambers, while 10% FBS-cultured medium (500 μL) was added into the lower chamber. After overnight culture, the remaining cells on the upper layer were erased. The invasive/migratory cells were fixed with formaldehyde and stained with crystal violet. Finally, the number of transmembrane cells was counted under a microscope.

### 4.7 Cell invasion assay

Cell invasion assays were performed using Matrigel-coated Transwell chambers (8 μm pore size; Costar). A total of 100 μL transfected cell suspension (2 × 10^4^ cells/mL) was added to the upper chamber of the Transwell plates. A total of 500 μL complete medium was added to the bottom chambers. After incubation for 24 h at 37 °C, nonmigrating cells were removed using cotton swabs. Cells that had permeated the Matrigel-coated membrane and migrated to the bottom were immobilized with paraformaldehyde and then stained with crystal violet.

### 4.8 Wound healing assay

Cells were seeded in 6-well plates at a density of 4 × 10^5^ cells/well. Once the cells reached 90% confluence, a wound area was carefully created by scraping the cell monolayer with a sterile 200 μl pipette tip from one end to the other end of the well. The detached cells were removed by washing them with PBS. Cells that migrated to the wounded region were observed by an Olympus inverted microscope and photographed (100× magnification) at 0 h and 24 h.

### 4.9 Western blotting

Cells were dissolved in RIPA buffer (Beyotime Biotechnology) containing protease inhibitors for the extraction of total protein, and protein quantification was then measured by a BCA Protein Assay Kit (Beyotime Biotechnology). Proteins (50 μg) were exposed to 10% SDS‒PAGE and electroplated onto a PVDF membrane (EMD Millipore, United States). The membranes were incubated overnight at 4 °C with the indicated primary antibodies (1:1000 dilution; all from Sangon Biotech): SIK1, E-cadherin, N-cadherin, Snail, FN, PAI-1, and GAPDH. Smad7 was purchased from Proteintech (China). Secondary antibodies diluted at 1:2000 were subsequently used for additional incubation at room temperature for 1h. The protein bands were visualized using the enhanced chemiluminescence method (Thermo Fisher Scientific, China).

### 4.10 Co-IP assay

HCT116 cell extracts were incubated for 2 h at 4 °C with IgG (Signalway Technologies, China) and protein A þ G Agarose (BEAVER, China) to eliminate nonspecific binding. SIK1 or Smad7 was then added at 4 °C overnight. The protein A/G-agarose was collected by centrifugation. Immunoprecipitated proteins were analyzed by SDS‒PAGE (10%, Minigel) at 100 V for 1.5 h. Membranes were blocked. SIK1 or Smad7 antibodies were diluted and incubated with membranes at 4 °C overnight. The secondary antibodies were then incubated at room temperature for 1 h. Western blots were visualized using enhanced chemiluminescence.

### 4.11 Inhibition experiments

The TGF-β signaling inhibitor galunisertib (LY2157299, GlpBio Technology, China) was administered at 10 μmol/ml 24 h after transfection with SIK1 siRNA, and then the transfectants were subjected to western blotting, transwell assays, and wound healing assays.

### 4.12 Immunohistochemical (IHC)

The cancerous tissue was dewaxed in xylene solution for 15 min. Then, 100% ethanol soaking, 95% ethanol soaking, 80% ethanol soaking, and 70% ethanol soaking were applied for 5 min each. Then, antigen repair was carried out at high temperature and high pressure for 5 min. Peroxidase inhibitor was applied at 37 °C for 20 min. The primary antibody was incubated at 4 °C overnight. The secondary antibody was added and incubated at room temperature for 1 h. DAB chromogenic solution was developed under an electron microscope, hematoxylin was stained for 5 min, and ethanol hydrochloride was differentiated for 1 min. Resin and cover glass were used to seal the film and photographed under an electron microscope. The staining scores were as follows: - for negative, + for weakly positive, ++ for moderately positive, and ++ for strongly positive. - or + was considered low expression, and ++ or +++ was considered high expression. Antibodies and dilutions were as follows: Ki67 (1:250, D220861, Sangon Biotech, Shanghai, China).

### 4.13 Mouse model and resistance to chemotherapy

To evaluate the role of SIK1 in CRC, a mouse model was established. In brief, female BALB/c nude mice (6-8 weeks old) were purchased from Guangdong Medical Laboratory Animal Center (Guangzhou, China) and bred under specific pathogen-free (SPF) conditions. The in vivo study was approved by the Ethics Committee of Guangdong Provincial People's Hospital (Guangdong Academy of Medical Sciences) under grant number GDREC2019506A. To establish a CRC mouse model, approximately 1 × 10^7^ HCT116shNC or HCT116shSIK1 cells were subcutaneously injected into the right flank. The protocol of grouping and therapeutic protocol can be found in Figure 7A. The mice were randomly divided into the following groups: the control group (phosphate buffer saline, PBS) and OXA group (5 mg/kg) after the tumor volume reached less than 150 mm3. PBS and OXA were administered intravenously 6 times every three days. The tumor volume was defined as follows: Volume = length × width^2^ × 0.5 mm^3^.

### 4.14 Statistical analysis

Statistical analyses were performed using GraphPad Prism version 8.0 (GraphPad Software, La Jolla, CA, United States) software and verified by SPSS version 26.0 (SPSS, Chicago, IL, United States). Each experiment was performed at least in triplicate, and the results are expressed as the mean ± standard deviation (SD). Student's t test and one-way analysis of variance were conducted to analyze the differences between groups, *p* < 0.05 was considered statistically significant, and *p* > 0.05 was considered not statistically significant (n.s.).

## 5. Conclusion

We demonstrated that SIK1 inhibits CRC metastasis and restores chemotherapy resistance by binding to Smad7 and inhibiting the TGF-β pathway. In summary, SIK1 can act as a tumor suppressor in colon cancer by inhibiting the TGF-β pathway. This study provides a first look at the tumor suppressive effects of SIK1 in CRC, complementing our previous work and providing new directions for CRC treatment by investigating this specific pathway in greater depth.

## Supplementary Material

Supplementary figures.
