# Supplementary material for: SIK1 suppresses colorectal cancer metastasis and chemoresistance via the TGF-β signaling pathway
Source: J Cancer. 2023 Aug 6;14(13):2455–67. doi: 10.7150/jca.83708 (PMC10475365; doi:10.7150/jca.83708)
Supplement: Supplementary file 1 — Supplementary figures. [file jcav14p2455s1.pdf]

Supporting Information for

SIK1 suppresses colorectal cancer metastasis and chemoresistance via the TGF-β signaling pathway

Yuan Gao [a, b, c, #], Hongming Li [a, b, c, d, #], Ping Wang [a, b, c, #], Junjiang Wang [a, b, *], Xueqing Yao [a, b, c, *]

[a] The Second School of Clinical Medicine, Southern Medical University, Guangzhou 510000, China

[b] Department of Gastrointestinal Surgery, Department of General Surgery, Guangdong Provincial People’s Hospital, Guangdong Academy of Medical Sciences, Southern Medical University, Guangzhou 510000, China.

[c] Department of General Surgery, Guangdong Provincial People's Hospital Ganzhou Hospital (Ganzhou Municipal Hospital), Ganzhou, 341000, China

[d] Department of colorectal surgery, Guangdong Provincial Hospital of Chinese Medicine, Guangzhou, 510120, China.

[#]These authors contributed equally to this work.

[*]To whom correspondence should be addressed.

Prof. Xueqing Yao, Email: syyaoxueqing@scut.edu.cn

Prof. Junjiang Wang, Email: sywangjunjiang@scut.edu.cn

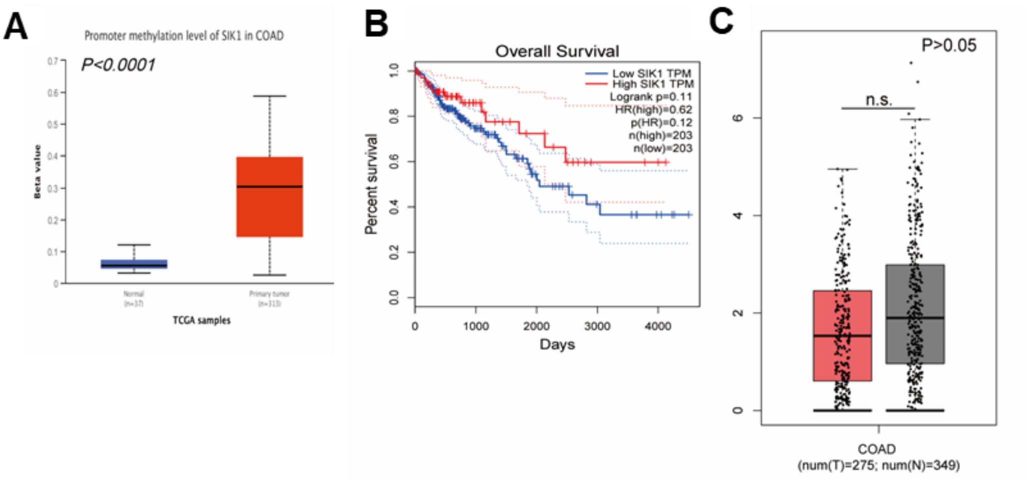

Figure S1 | The bioinformatic analysis of SIK1.

(A) SIK1 promoter methylation from TCGA database, CRC tissues compared with that in normal colon tissues by data analysis.

(B) SIK1 The relationship about Overall Survival between the expression of SIK1 and CRC patients from GEPIA.

(C) The expression of SIK1 on CRC patients between the CRC tissues and paired normal colorectal tissues from GEPIA.

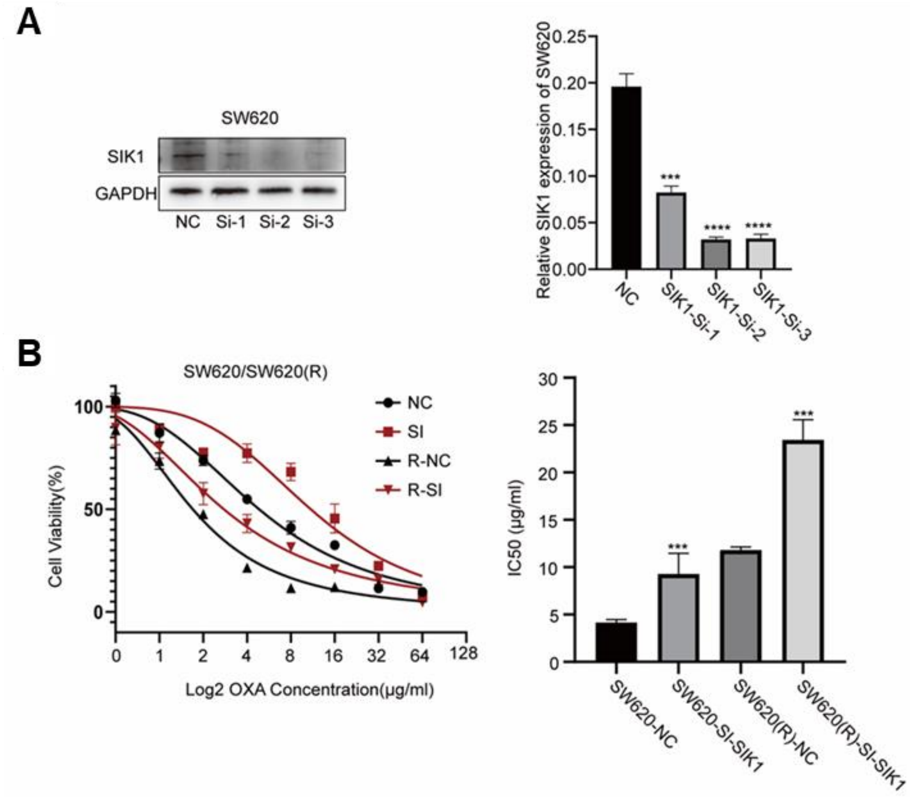

Figure S2 | The validation analysis of SW620(R) chemoresistant cell line.

(A) Western blot analysis was performed for SIK1 in SW620 cells transfected with SIK1-Si-1, SIK1-Si-2, and SIK1-Si-3. S2(B) The knockdown of SIK1 significantly decreased sensitivity to OXA in normal CRC cell lines (SW620) and OXA-resistant cell lines (SW620(R)). Statistical analysis was performed in triplicate experiments via unpaired Student's t test. Data are expressed as the mean ± SD (n=3), *n.s.* p>=0.05, *$p<0.05$, **$p<0.01$, *** $p<0.001$, ****$p<0.0001$.

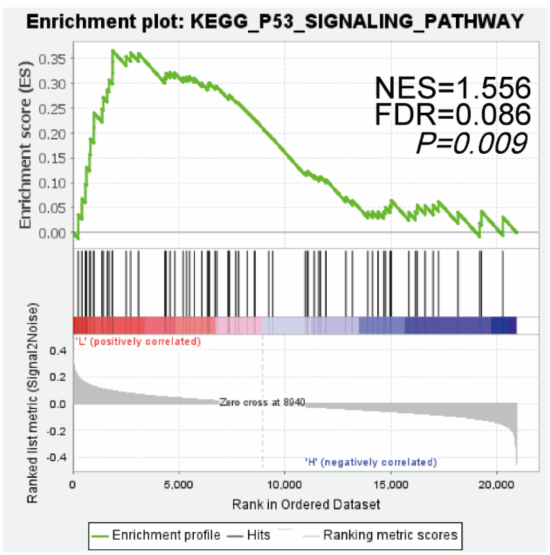

Figure S3 | GSEA KEGG pathway enrichment analysis in the P53 signaling pathway. NES>1, FDR<0.25, **p<0.01 considered significant.

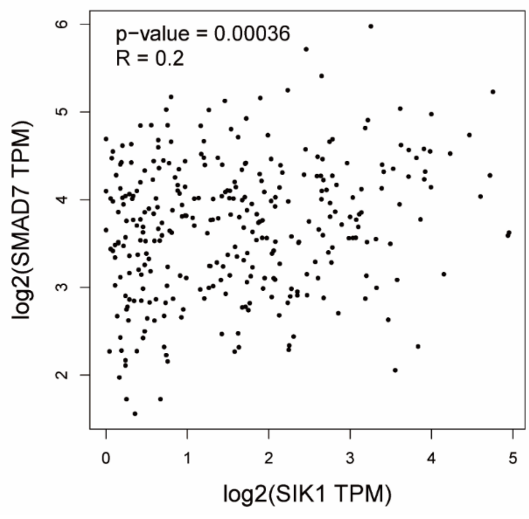

Figure S4 | GEPIA bioinformatics analysis showed that SIK1 was positively correlated with SMAD7
